# Mechanisms of first-line treatment resistance in diffuse large B-cell lymphoma

**DOI:** 10.3389/fimmu.2026.1776324

**Published:** 2026-05-08

**Authors:** Zhumei Zhan, Peipei Li, Yanlu Liu, Changqing Zhen, Ying Zhang, Ou Bai

**Affiliations:** 1 Department of Hematology, Shandong Provincial Hospital Affiliated to Shandong First Medical University, Jinan, Shandong, China; 2 College of Food and Pharmaceutical Engineering, Zhaoqing University, Zhaoqing, Guangdong, China; 3 Department of Hematology, Beijing Friendship Hospital Affiliated to Capital Medical University, Beijing, China; 4 Department of Hematology, The First Hospital of Jilin University, Changchun, China

**Keywords:** chemotherapy, diffuse large B-cell lymphoma, drug resistance, rituximab, virus

## Abstract

Diffuse large B-cell lymphoma (DLBCL) is the most common subtype of malignant lymphoma in adults. Although 60–70% of patients achieve remission with first-line immunochemotherapy (R-CHOP or pola-R-CHP), approximately 30%-40% of patients still experience develop refractory or relapsed disease. Treatment resistance still remains as the major challenge in DLBCL patient management. Although there is no significant synergy among the components in the R-CHOP or Pola-R-CHP regimens, cross-resistance between these treatments is minimal. Elucidating the mechanisms underlying drug resistance and developing innovative therapeutic strategies are essential for enhancing the clinical outcomes for this patient cohort. This article provides a detailed and up-to-date review of the molecular mechanisms of resistance to the current treatment options in DLBCL. We lastly highlight potential strategies for the rational management of treatment resistance.

## 1 Introduction

Diffuse large B-cell lymphoma (DLBCL) is the most common subtype of lymphoma in adults, comprising approximately 25% of the total non-Hodgkin lymphoma (NHL) cases in the United States and typically more than 40% in Asian countries (1–3). An epidemiological study of 10, 002 cases collected from 24 medical centers in China reported that DLBCL accounted for 45.8% of all NHLs and 40.1% of all lymphoma subtypes (4). First-line DLBCL treatment involves a combination of DNA damaging agents (cyclophosphamide, adriamycin and vincristine) and the synthetic glucocorticoid prednisolone, together with rituximab. The five drugs comprising R-CHOP did not show synergistic interactions but very low cross-resistance, indicating that the efficacy of R-CHOP comes from combination therapy with active agents with non-overlapping resistance mechanisms (5). This consideration allows us to interrogate molecular mechanisms of treatment resistance by evaluating tumor responses to the individual R-CHOP components.

Polatuzumab vedotin is another antibody-drug conjugate that targets the CD79b antigen expressed on lymphocytes, and it has been approved for first-line treatment of DLBCL. The recently published POLARIX trial also showed that replacing vincristine with polatuzumab in front line R-CHOP (pola-R-CHP) for patients with DLBCL led to a higher rate of 2 year progression free survival (PFS) but not overall survival (OS) as compared to R-CHOP (6). Although the addition of rituximab has led to a significantly increased survival of patients with DLBCL, with a 10-year PFS rate of 43.5% (7). However, 30-40% of patients still develop refractory disease or relapse following initial remission, with primary and acquired resistance accounting for the main reasons. The clinical prognosis for this patient cohort is usually unfavorable, with a median OS of 6.3 months and a 1-year OS rate of 28% (8). This review summarizes the current knowledge of potential mechanisms associated with rituximab-, polatuzumab vedotin- and chemotherapy-resistant DLBCL and aims to provide an integrated framework of relevant targets for therapeutic intervention of drug-resistant DLBCL.

## 2 Polatuzumab vedotin-resistance

A single-center retrospective analysis demonstrated that high grade B cell lymphoma- not otherwise specified (NOS) or MYC/BCL2 histology and/or MYC rearrangement as predictors of inferior response to polatuzumab based therapy (9). In a separate study using flow cytometry to assess CD79b cell surface expression, it was found that a minimum threshold of 6.82 geometric mean fluorescence intensity units of CD79b expression is required for effective anti-CD79b antibody-dependent cellular cytotoxicity (ADCC) (10). Downregulation of CD79b expression represents the primary mechanism of resistance to polatuzumab vedotin. Kawasaki et al. explored possible mechanisms of resistance to polatuzumab vedotin in a panel of DLBCL cell lines and identified three factors low CD79B surface expression, high expression of ATP binding C cassette (ABC) transporters (multidrug resistance pumps), and high expression of the anti-apoptotic protein Bcl-xL (BCL2L1) (11). RNA seq data showed that Dehydrogenase 1 Family Member L1 (ALDH1L1) is the second most upregulated gene in polatuzumab vedotin-resistant cell (12). Previous studies indicated that the deregulation of ALDH1L1 can disrupt various cellular metabolic pathways, potentially reducing dependency on B cell receptor (BCR) signalling and CD79B expression, leading to reduced sensitivity to polatuzumab vedotin (13). A subset analysis of the POLARIX clinical trial revealed a benefit of polatuzumab vedotin in ABC but not GCB DLBCL. Notably, the lower expression of KLHL6 in ABC DLBCL may have contributed to its susceptibility to polatuzumab vedotin. The efficacy of polatuzumab vedotin is likely to be multifactorial and influenced by genetic and epigenetic factors that regulate BCR expression on the cell surface, such as KLHL6, as well as the accessibility of the polatuzumab binding epitope on CD79B, which is modulated by N-linked glycosylation (14). Research by Elisabeth A. Lasater et al. demonstrated that the antibody-drug conjugate polatuzumab vedotin promotes MCL-1 degradation via the ubiquitin-proteasome system. This targeted MCL-1 antagonism, when combined with venetoclax and anti-CD20 antibodies obinutuzumab or rituximab, induces tumor regression in preclinical DLBCL models, which are sustained even off-treatment (15).

## 3 Rituximab-resistance

**Figure 1:**
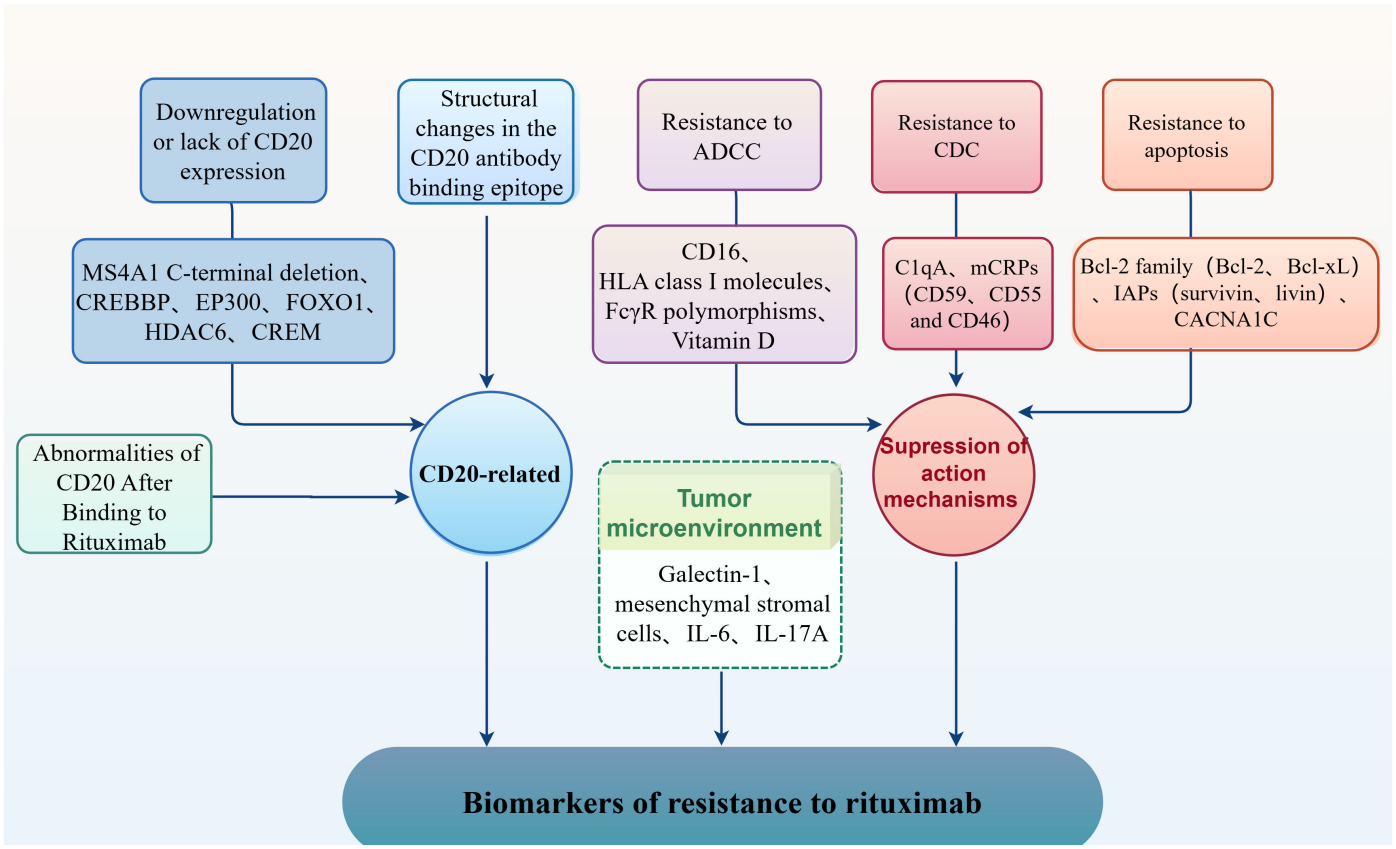
Biomarkers of resistance to rituximab.

Rituximab, a human-mouse chimeric anti-CD20 monoclonal antibody, is crucial in treating mature B-cell malignancies (16). The CD20 antigen, whose expression is restricted to B-cell lines, is encoded by the MS4A1 gene located on chromosome 11q12. Rituximab initiates an immune response mediating B cell lysis by specifically binding to the CD20 antigen through three mechanisms: antibody-dependent cellular cytotoxicity (ADCC), complement-dependent cytotoxicity (CDC), and direct induction of apoptosis (17). Although rituximab is a landmark drug in the treatment of DLBCL, not all DLBCL patients will benefit from it. As indicated in **Figure 1**, rituximab resistance is closely associated with the transcriptional and translational processes of the MS4A1 gene, alterations in the structure of the CD20 antibody-binding site or rearrangement of the structural domains of lipid rafts, and resistance to above three mechanisms of action. Additionally, the PTEN/PI3K/AKT (18) and BCR (19)signaling pathway, which is intricately linked to the progression of DLBCL, is involved in the response to rituximab in certain individuals.

### 3.1 Downregulation or lack of CD20 expression

A study by Terui et al. (20) reported that in 50 patients with relapsed/resistant NHL (DLBCL, n=22), 11 (22%) were detected with *MS4A1* gene C-terminal deletion mutations. In addition, CD20 antigen expression is co-regulated by a variety of epigenetic regulators and transcription factors (21, 22). Studies have demonstrated that histone acetyltransferase depletion (CREBBP/EP300) reduces CD20 expression by impairing transcription factor PU.1 binding to the CD20 promoter (23). Activating mutations in FOXO1 enhance its binding to the MS4A1 promoter, suppressing CD20 transcription (24). HDAC6 represses CD20 transcription, while HDAC inhibitors enhance anti-CD20 monoclonal antibody efficacy (25). Genome-wide RNAi screening identified CREM as a key CD20 suppressor, with CHD4 and MBD2—components of the Nucleosome Remodeling Deacetylase complex—acting as transcriptional activators of CD20 (26). Additionally, PDK4 negatively correlates with CD20 expression by transcriptionally repressing it, thereby modulating DLBCL cell sensitivity to rituximab (27).

### 3.2 Structural changes in the CD20 antibody binding epitope

Other transcriptional splice variants of CD20 exist in malignant B-cell lymphomas, some of which encode truncated forms of the CD20 antigen, which bind weakly to anti-CD20 monoclonal antibodies. Henry et al. (28) reported that aberrant expression of CD20 mRNA splice variants has a conformational change in the rituximab binding epitope and an alteration of the localization of CD20 on the membrane, contributing to the development of drug resistance.

### 3.3 Resistance to ADCC

ADCC refers to the direct killing of tumor cells by cells with cytotoxic activity (e.g., natural killer cells, macrophages, and neutrophils) through recognizing encapsulated Fc fragments located on target antigen by Fc receptors (FcγR) on their surface. Defective NK-cell activation rather than target antigen loss potentially contributes to resistance (29). Impaired ADCC in DLBCL may result from low CD16 expression on NK cells, reducing degranulation upon encountering rituximab-coated tumor cells (30). Fc gamma receptor (FcγR) single nucleotide polymorphisms (SNPs) are associated with affinity for rituximab. The most commonly polymorphism is FcγR IIIA, compared to those with 158V/F or 158F/F, cells with FcγR IIIA 158V/V have a higher affinity for the IgG *in vitro* (31, 32). Additionally, high HLA class I expression on tumor cells can inhibit NK function, contributing to treatment failure (33). Vitamin D3 deficiency worsens outcomes in rituximab-treated patients, primarily by attenuating ADCC effects (34, 35).

### 3.4 Resistance to CDC

Complement-dependent cytotoxicity (CDC) is initiated when complement C1q binds to the antibody Fc segment, activating the classical pathway to lyse target cells. *In vitro* and *in vivo* studies confirmed that rituximab resistance has been linked to downregulation of C1qA, regulated via METTL3/YTHDF2-mediated m6A methylation (36). However, Keane C et al. showed that C1qA-A276G polymorphism did not correlate with survival outcomes in DLBCL patients treated with rituximab (37). Another Japanese study (38) reported that rituximab-resistant cells expressed higher membrane complement regulatory proteins (mCRPs), such as CD59, CD55, and CD46, however, mCRPs blockade have limitations in clinical application as they play a key regulatory role in protecting normal cells from excessive complement-mediated cytotoxicity. While serum complement’s exact role remains debated, excessively applied rituximab can deplete complement (39), and exogenous complement supplementation has been explored to enhance CDC effect of rituximab.

### 3.5 Resistance to apoptosis

Rituximab-resistant lymphoma cell lines demonstrate constitutive hyperactivation of NF-κB, MAPK, and PI3K/AKT pathways, leading to overexpression of anti-apoptotic proteins such as Bcl-2/Bcl-XL (40) and IAP family members survivin/livin (41). Future clinical studies should focus on restoring sensitivity to rituximab-mediated apoptosis through the combined application of targeted anti-apoptotic molecular inhibitors. Calcium influx is an important inducer for rituximab-induced apoptosis through affected mitochondrial depolarization and activated the apoptosis executor cysteine asparaginase (42). Loss of the L-type calcium channel α1C (CACNA1C) reduced rituximab-induced apoptosis and CACNA1C was directly regulated by miRNA-363 whose high expression is associated with worse prognosis in DLBCL (43).

### 3.6 Tumor microenvironment

Interactions between the tumor microenvironment (TME) and malignant B cells play a crucial role in disease progression, immune escape, and treatment resistance. Gene expression profiling identified favorable (extracellular matrix deposition, histiocytic infiltration) and unfavorable (high tumor blood-vessel density) TME signatures associated with the efficacy of rituximab-containing therapy (44). Lykken et al. (45) found that increased galectin-1 (Gal-1) expression within the microenvironment impairs ADCC and reduces response to CD20 immunotherapy *in vivo*. In recent years, there has been extensive research conducted on the involvement of mesenchymal stromal cells (MSCs) in the tumor microenvironment. MSCs have been found to play a dual role: they downregulate CD20 expression on B cells and protect malignant cells from apoptosis via stromal adhesion (46). Despite the rarity of bone marrow infiltration in DLBCL, an *in vitro* study revealed that bone marrow-derived MSCs promote resistance through IL-6 and IL-17A in microenvironment (47). Zhong W et al. found that rituximab promotes Th17 and IL-17+Foxp3+ Treg cells to secrete IL-17A, which partially by suppressing p53 expression and inhibiting rituximab-induced apoptosis (48).

## 4 Chemotherapy-resistance

**Figure 2:**
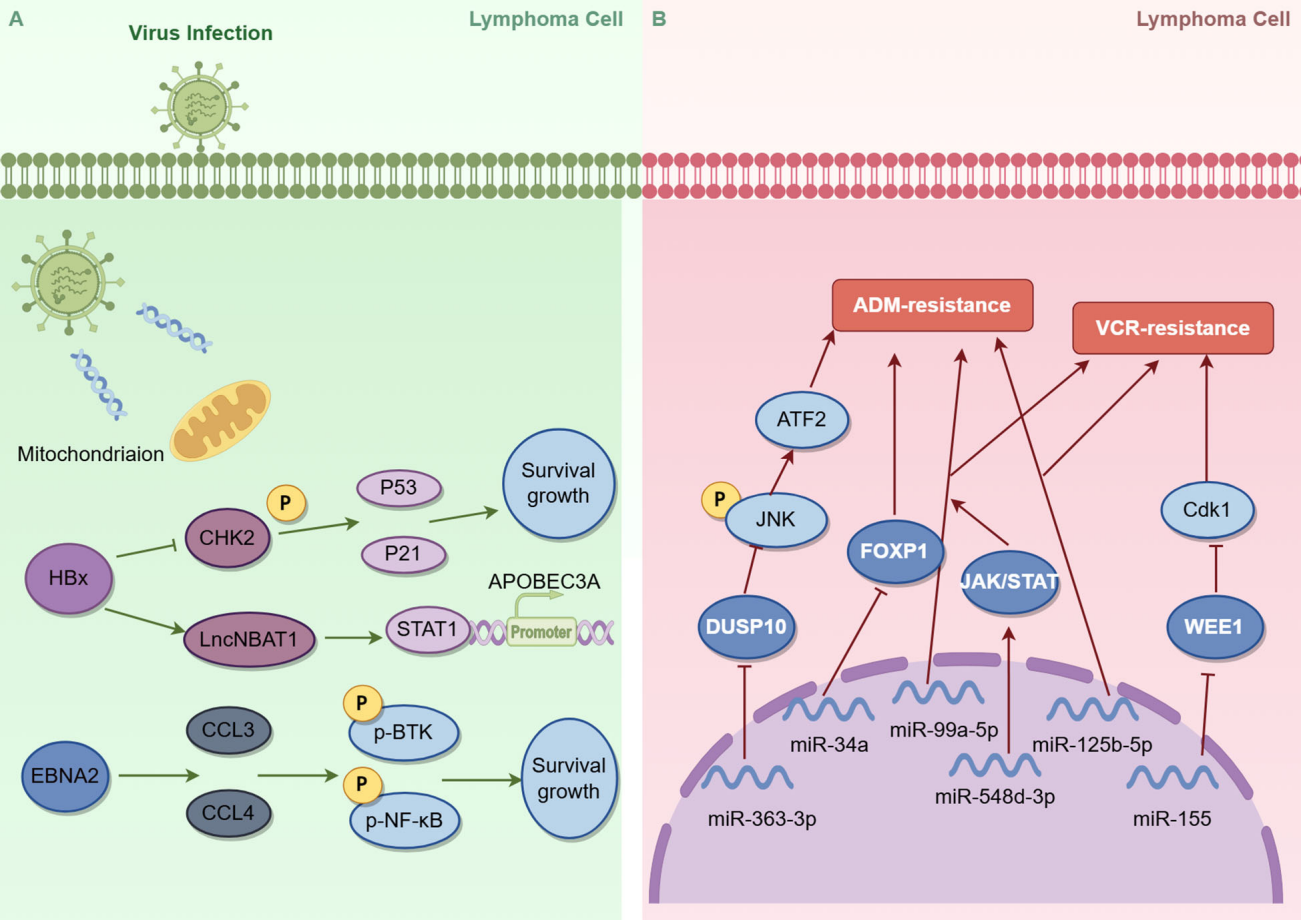
Role of viral infection **(A)** and miRNA **(B)** in DLBCL chemoresistance

Chemotherapy resistance relates to upregulated multidrug resistance genes, enhanced DNA repair, apoptosis resistance, TME alterations, miRNA dysregulation, and genomic abnormalities (**Figure 2**). Glucocorticoid resistance specifically involves suppressed glucocorticoid receptor (GR) expression, signaling pathway interference, and altered transcriptional co-regulators recruitment (49). Recent research has revealed a strong association between viral infections, particularly hepatitis B virus (HBV) and Epstein-Barr virus (EBV), and the pathogenesis of DLBCL. Viral infections, particularly HBV and EBV, are also implicated in chemoresistance by inducing DNA damage responses and activating oncogenic pathways (50, 51).

### 4.1 Gene mutations

The hallmark of DLBCL is characterized by the occurrence and accumulation of genetic alterations that drive malignant B-cell survival, a survival advantage that may be influenced by selection pressure during treatment, leading to the development of chemoresistance. A study comparing mutation profiles of patients with relapsed/refractory DLBCL (n=25) to those with primary DLBCL (n=138) (52), indicated that *TP53*, *FOXO1*, *MLL3 (KMT2C)*, *CCND3*, *NFKBIZ*, and *STAT6* were mutated at a higher frequency during relapse, potentially undergoing clonal amplification after immunochemotherapy and becoming candidate genes associated with treatment resistance. Another large sample study showed that *TP53* and *KMT2D* were mutated in the majority of 135 relapsed/refractory DLBCL, and these mutations remained clonally persistent throughout treatment in paired diagnostic-relapse samples, suggesting a role in primary treatment resistance (53). Given the pivotal role of TP53 mutations in driving primary treatment resistance and disease relapse, their prognostic utility in DLBCL has garnered substantial research interest. Notably, recent evidence has uncovered significant site-specific prognostic heterogeneity among TP53 hotspot mutations. Patients harboring mutations in high-risk residues (G245, R175, R248, R273, R282) exhibited markedly inferior PFS and OS, whereas those with low-risk mutations (C242, Y234, Y236) demonstrated outcomes comparable to TP53 wild-type counterparts. When patients in the high-risk group received R-CHOP plus targeted therapy, their survival outcomes were comparable to those of TP53 wild-type patients (54).

### 4.2 Epigenetic

*NOTCH2* mutations evade ubiquitin-dependent degradation by E3 ligases KLHL6 and FBXW7, leading to activation of the RAS/AKT pathway and contributing to resistance to CHOP regimen in DLBCL (55). USP1 and USP28, members of the ubiquitin-specific processing enzyme family, have been identified in DLBCL chemoresistance (56, 57). USP1 stabilizes the MAX/MYC heterodimer via deubiquitination, sustaining MYC oncogenic transcription. Sirtuin1 (Sirt1), NAD+-dependent histone deacetylases, is instrumental in promoting adriamycin resistance in DLBCL through the activation of mitochondrial energy metabolic pathways. Furthermore, *in vivo* studies have shown that chemoresistance induced by Sirt1 overexpression can be reversed by combining with the mitochondrial energy inhibitor tigecycline (58). Moreover, high expression of Sirt6 expression has been associated with unfavorable prognosis in patients with DLBCL. Experiments conducted *in vitro* have demonstrated that DLBCL cells exhibited enhanced chemosensitivity following inhibition of Sirt6 expression, as indicated by increased apoptosis, disrupted cell proliferation, and arrest in the G2 and M phases of the cell cycle following chemotherapy (59).

### 4.3 Inflammation and CSCs of the TME

ADAM-12 is a multifunctional protein involved in a variety of biological processes, including cell adhesion. A study showed that highly expressed ADAM12 promotes cell adhesion-mediated adriamycin resistance via AKT signaling pathway in DLBCL cells (60). Lv et al. (61) demonstrated that the secretion of IL-9 by activated Th2 cells in the TME confers protection to DLBCL cells against chemotherapy-induced apoptosis. Cancer stem cells (CSCs) in TME play a crucial role in disease progression, recurrence, metastasis, and treatment resistance. Chen et al. (62) revealed a notable increase in the proportion of CSCs in chemotherapy-resistant DLBCL cell lines, attributed to the up-regulation of the stem cell-associated transcription factor SRY-box 2 (SOX2). SOX2 can be phosphorylated by the activated PI3K/AKT signaling pathway, leading to the inhibition of its ubiquitin-mediated degradation. Thus, the PI3K/AKT/SOX2 axis has been identified as a key player in reversing chemoresistance in DLBCL through the down-regulation of SOX2. Another study has shown that the CircPCBP2/miR-33a/b/PD-L1 axis may enhance stem cell properties in DLBCL and reduce sensitivity to CHOP, suggesting a potential therapeutic target for treatment-resistant DLBCL (63).

### 4.4 Metabolomics

As lymphomas progress, the nutrient availability within the TME fluctuates, prompting lymphoma to sustain their rapid proliferation and survival through alterations in lipid metabolism. These changes in lipid metabolism have been shown to impact the efficacy of chemotherapy. Barre et al. studied differences in the lipid and metabolic composition of drug-resistant DLBCL, using a combination of xenograft models and mass spectrometry imaging, which were mainly characterized by reduced adenosine triphosphate and increased adenosine monophosphate (64). These findings systematically elucidate the multidimensional regulatory network underlying DLBCL chemotherapy resistance, providing new directions for developing combined therapeutic strategies targeting the metabolism. For the high-risk DLBCL cases with a strong MYC-driven expression signature, the selection of some key Amino Acid influenced by MYC, such as Gln, glutamate, tyrosine, tryptophan, and BCAAs, correlating with a poor response to standard therapies (65).

### 4.5 miRNA

miRNAs serve as mediators between lymphoma cells and the microenvironment, modulating signaling pathways involved in chemoresistance. Marques et al. (66) performed miRNA expression profiling and drug-response screening in 15 human DLBCL cell lines. Their findings indicated that high expression of miRNA-34a was correlated with increased susceptibility to adriamycin in DLBCL, potentially through the down-regulation of its target FOXP1. The relationship between high FOXP1protein expression and adriamycin resistance was identified previously (67). Due et al. (68) identified 15 differentially expressed miRNAs in vincristine-resistant DLBCL, most notably downregulated miRNA-155, which promoted resistance by increasing WEE1 expression, inhibiting Cdk1 activity, and arresting cells at the G2/M checkpoint. Zhou et al. (69) conducted *in vitro* and *in vivo* experiments demonstrated that miRNA-363-3p upregulated JNK phosphorylation through the downregulation of dual-specificity phosphatase 10 (DUSP10), leading to the repair of adriamycin-induced DNA double-strand breaks and inhibited adriamycin-induced apoptosis (**Figure 2**). Downregulation of miRNA-548d-3p has been linked to chemoresistance in DLBCL patients through BCL2 upregulation, JAK/STAT activation, and p53 pathway disruption (70). Exosomal miRNA analysis in 116 DLBCL patients revealed elevated miRNA-99a-5p and miRNA-125b-5p in resistant cases, with further studies implicating the AMPK, TGF-β, mTOR, and p53 signaling pathway in exosome-mediated chemoresistance (71).

### 4.6 Drug transport and metabolism

Chemotherapy resistance in DLBCL is often driven by ATP-binding cassette (ABC) transporters, including P-gp (ABCB1), MRP1 (ABCC1), and BCRP (ABCG2), which efflux drugs like adriamycin and vincristine, reducing intracellular accumulation (72). Adriamycin and vincristine are considered substrates of P-gp, and the application of these drugs also induces up-regulation of P-gp expression in patients with DLBCL (73). Clinical studies have demonstrated a lower response to induction chemotherapy in DLBCL patients with expression of P-gp or MRP1 (74). However, targeting ABC transporters is clinically challenging due to their essential physiological roles and potential toxicity. Liu et al. (75) conducted a proteomic analysis of CHOP-resistant versus sensitive DLBCL samples identified 19 differentially expressed proteins, with approximately 20.6% of them being implicated in drug metabolism pathways, notably GSTP1 and ALDH1A1. GSTP1 promoter hypermethylation is involved in resistance to agents like adriamycin (76).

### 4.7 DNA damage repair

The DNA damage response determines the sensitivity to chemotherapeutic agents to a certain extent (77). High-risk patients with DLBCL are characterized by significant enrichment of genes encoding for nucleotide excision repair (NER) pathway in tumor samples, including ERCC2/XPD, ERCC3/XPB, ERCC4/XPF, ERCC6/CSB, and ERCC8/CSA, DDB2 and polymerase delta (78). SAMHD1 has a dNTPase-independent role in promoting resection to facilitate DNA double-strand break repair by homologous recombination. Low SAMHD1 expression has been confirmed associated with higher sensitivity to adriamycin and PARP inhibitors in DLBCL (79).

### 4.8 Apoptosis

While multiple mechanisms contribute to chemoresistance in DLBCL, they ultimately converge on the suppression of apoptosis—the primary mode of cell death induced by most chemotherapeutic drugs. Elevated expression of apoptosis inhibitors correlates with unfavorable clinical prognosis in DLBCL (80, 81). Cillessen et al. demonstrated that chemotherapy-resistant DLBCL samples show increased expression of both pro- and anti-apoptotic genes, including BCL-2 family (Bax, Bak, Bad, Puma, and Bid) as well as the IAP family (cIAP2, XIAP, NIAP, and Apollon) (82). Functional studies revealed that RelB confers DLBCL cell resistance to DNA damage-induced apoptosis in response to doxorubicin, RelB positivity is associated with high expression of cIAP2 (83).Targeting XIAP with small-molecule antagonists restore downstream caspase-3/7 activity and enhances chemotherapy-induced apoptosis, highlighting IAPs as promising therapeutic targets to overcome resistance (84).

### 4.9 Viral infection

China is recognized as a region with a high prevalence of Hepatitis B Virus (HBV) infection, with epidemiological studies revealing that between 11.6% and 30.9% of DLBCL patients test positive for Hepatitis B surface antigen (HBsAg) (85–88). As summarized in **Table 1**, HBsAg-positive patients demonstrated a tendency toward lower CR rates and shorter OS undergoing first-line chemotherapy compared with HBsAg-negative DLBCL patients. A prospective, multicenter, non-interventional study suggests that HBsAg positivity serves as an independent risk factor affecting the achievement of CR with first-line chemotherapy in Chinese DLBCL patients. An experimental study has shown that HBx, a functional protein encoded by HBV, specifically inhibits CHK2 (a critical DNA damage response protein) phosphorylation, thereby inducing resistance in DLBCL cells to S-phase arrest drugs such as second-line chemotherapeutic agents (MTX, Ara-C) (50). Subsequent investigations conducted by the same research team uncovered that HBx directly upregulates the expression of lncNBAT1, which interacts with STAT1, preventing its enrichment in the promoter region of APOBEC3A and inhibiting APOBEC3A expression, leading to the resistance of DLBCL cells to MTX and Ara-C (89) (**Figure 2**). APOBEC3A is identified as a crucial target of lncNBAT1 in relation to chemoresistance, as well as a significant contributor to the increased mutation rate observed in the genome of HBsAg-positive DLBCL (90).

**Table 1:** Summary of clinical studies on the effect of HBV infection on outcomes in patients with DLBCL.

Author	Number of patients			Treatment	Response to chemotherapy
	Total	HBsAg-positive	HBsAg- negative		
Wang F (85)	362	81	181	R ± CHOP ± RT	ORR: 91.0% vs. 90.4%; CR: 53.0% vs. 61.7%; Median OS: 55.8m vs. 66.8m
Xie W (97)	451	90	361	R ± CHOP	OS rate: 62.2% vs. 76.2%
Wei Z (98)	384	56	328	R ± CHOP	ORR: 91.1% vs. 88.1%; CR: 73.2% vs. 69.5%
Deng L (86)	587	81	506	R ± CHOP	ORR: 55.5% vs. 80.0%; CR: 37.0% vs. 64.4%; 2-year OS: 47.0% vs. 70.0%; 2-year PFS: 36.0% vs. 61.0%
Law MF (99)	81	16	65	R ± CHOP ± RT	ORR: 75.0% vs. 65.0%; CR: 63.0% vs. 54.0%
Liu WP (100)	107	46	61	R ± CHOP	ORR: 71.8% vs. 86.9%; CR: 45.7% vs. 57.4%; Median OS: 24.5m vs. 26.3m; Median PFS: 14.7m vs. 20.5m
Liu Z (101)	81	30	51	R ± CHOP	Median OS: 38.6m vs. NR; Median PFS: 18.5m vs.38.5m
Zhao X (50)	428	93	335	–	ORR:61.3% vs. 73.4%
Wang Y (102)	472	113	359	R ± CHOP	ORR: 77.9% vs. 88.0%; CR: 27.4% vs. 61.3%
Guo YF (103)	246	80	166	RCHOP	ORR: 55.0% vs. 80.8%; CR: 36.2% vs. 63.9%
Kang X (87)	319	132	187	R ± CHOP	ORR: 97.0% vs. 95.7%; CR: 56.1% vs. 49.2%
Cheng CL (104)	416	98	318	R-CHOP	ORR: 76.5% vs. 85.5%; 5-year OS: 57.2% vs. 73.5%; 5-year PFS 47.2% vs. 60.7%
Chen DG (88)	420	127	293	R ± CHOP	ORR: 92.9% vs. 95.2%; CR: 61.4% vs. 66.9%; Median OS: 66.9m vs. 63.9m
Yamauchi N (105)	394	116	278	R-CHOP	ORR: 97.4% vs. 92.5%; CR: 89.7% vs. 83.8%; 4-year OS: 77.5% vs. 82.2%; 4-year PFS: 66.8% vs. 73.7%

ORR, Objective response rate; CR, Complete response; OS, Overall survival; PFS, Progression free survival; NR, not reached; RT, Radiation therapy.

The incidence of Epstein-Barr virus (EBV) infection in patients with DLBCL varies across different regions, with a lower prevalence observed in Europe and the United States (≤5%) compared to higher rates in Asian and Latin American countries (10%-15%) (91). EBV-positive DLBCL is recognized as a distinct subtype in the 2022 World Health Organization Classification of Tumors of Lymphoid Tissues, characterized by lower response rates with CR ranging from 30-60% when treated with CHOP regimen, and shorter OS (92, 93). Depletion or inactivation of chemokines CCL3 or CCL4 in DLBCL indicates increased sensitivity to adriamycin. The EBV-encoded nuclear antigen EBNA2 activates the BTK/NF-κB pathways by upregulating CCL3 and CCL4 expression, leading to resistance to adriamycin in lymphoma cells (94). Yoon et al. proposed that the EBV-encoded membrane protein LMP1 may contribute to the poor response to chemotherapy in EBV-positive DLBCL (95). Additionally, EBV-positive DLBCL cells demonstrate high expression of ABC transporter protein family members MDR1, MRP1, and MRP2 (96).

## 5 Rational management of treatment resistance in DLBCL

On one hand, predicting treatment response allows for the stratification of patients who are most likely to benefit from certain therapies, while sparing those who are refractory or may develop resistance from unnecessary exposure. Furthermore, real-time monitoring of treatment response enables dynamic risk assessment, facilitating timely adjustments to patient management to help prevent potential occurrence of resistance. On the other hand, several strategies have been employed to overcome treatment resistance. Early approaches primarily relied on chemotherapy regimens without cross-resistant drugs, whereas today immunotherapies and targeted drugs with novel mechanisms of action are employed. For instance, many preclinical and clinical studies have evaluated novel combinations of targeted therapies, epigenetic therapies, and immunotherapies to enhance treatment efficacy and to overcome resistance. Xia Z et al. used a combination of rituximab-based R-CHOP scheme and DNA tetrahedra to fabricate antibody-DNA nanostructure conjugate (ADNC), demonstrating a robust antitumor effect *in vitro*, significantly exceeding the combined effects of rituximab and doxorubicin by >50-fold (106). Chidamide plus R-GemOx demonstrated encouraging antitumor activity in transplant-ineligible patients with R/R DLBCL, achieving an ORR of 59.3% with acceptable toxicity (107). BTKi combined with chemotherapy, immunotherapeutic agents, and small-molecule inhibitors has achieved some progress in treating R/R DLBCL. A study evaluated the efficacy and safety of polatuzumab vedotin and zanubrutinib plus rituximab (Pola-ZR) or obinutuzumab (Pola-ZG) in patients with R/R DLBCL. The Pola-ZR/G treatment group achieved an ORR of 70%, significantly higher than that of conventional salvage therapy (108). Additionally, studies have explored the use of ibrutinib in combination with R-Gemox for non-GCB R/R DLBCL (109). The ORR for other small-molecule inhibitors such as CD47 inhibitor (Hu5F9-G4) (110), CUDC-907(small molecule inhibitor of both HDAC and PI3K) (109) in treating R/R DLBCL ranges from 30% to 40%. Anti-CD19 CART (axi-cel, liso-cel) led to significant improvements in R/R DLBCL, as compared with standard care, in event-free survival and response, with the expected level of high-grade toxic effects (111, 112).

## 6 Conclusion

Although progress has been made in understanding the mechanisms underlying first-line treatment for DLBCL, its clinical application remains limited. Accurate stratification of patients at diagnosis and early identification of those unlikely to respond to standard immunochemotherapy regimens, such as R-CHOP or pola-R-CHOP, are critical to improving outcomes in DLBCL. As research progresses, we anticipate integrating genetic, immunohistochemical, and serum biomarkers to reliably predict treatment response for DLBCL patients treated with first-line regimens. The increased understanding of DLBCL biology, tumor microenvironment, genetic subtypes, and epigenetics will bring more novel therapeutic strategies and gene-oriented therapeutic approaches.
